# *‘The eyes of others’ are what really matters*: The experience of living with dementia from an insider perspective

**DOI:** 10.1371/journal.pone.0214724

**Published:** 2019-04-03

**Authors:** Els van Wijngaarden, Manna Alma, Anne-Mei The

**Affiliations:** 1 Research Group Care Ethics, University of Humanistic Studies, Utrecht, The Netherlands; 2 Research organisation Tao of Care, Amsterdam, The Netherlands; 3 Department of Health Sciences, Applied Health Research, University Medical Center Groningen, University of Groningen, Groningen, The Netherlands; 4 Faculty of Social and Behavioural Sciences, University of Amsterdam, Amsterdam, The Netherlands; University of Toronto, CANADA

## Abstract

**Introduction:**

This Dutch study is a qualitative interview study. It aims to contribute to our understanding of the day-to-day experiences by providing an idiographic description of what it *means* existentially to be in the world as a person affected by a form of dementia, taking into account the contextual nature of these embodied experiences.

**Methods:**

We used a combination of narrative accounts of people from dementia. We first collected 322 recorded messages of 16 diarists who joined the Dutch Dementia Diaries project. This data was supplemented with 37 interview accounts. Our data analysis was inspired by Van Manen’s existential phenomenological approach.

**Results:**

The findings show that living with dementia–from a first-person perspective–can be understood as a severely unsettling experience: the people concerned enter a very uncertain, unpredictable and ambiguous period of life. They have to face all kinds of losses that considerably change and disrupt their relationships with 1) their own body, 2) with others and 3) with the surrounding world. This experience is explicated in the following themes: 1a) scrutinizing your disrupted body; 1b) trying to control your bodily loss-of-control; 2a) feeling scrutinized by the suspicious gaze of others; 2b) drifting away from significant others; 2c) having difficulties sharing the struggle; 2d) longing to be taken seriously; 2e) engaging in a world of peers; and 3a) sensing disorientation in an alien place; 3b) feeling closed in within a shrinking space; 3c) trying to control a dreaded future; 3d) trying to control a dreaded future by means of euthanasia.

**Discussion:**

Our study demonstrates how the people with dementia are affected by ‘the eyes of others’. They longed for a safe and accepting environment, but quite often felt scrutinized by inquisitive and disapproving looks. The outcomes also reveal a connection between dominant social imaginaries and people’s self-understanding of dementia. Much of the suffering stems from living under the shadow of negative imaginaries. Furthermore, our study supports the demand for a socio-relational approach by demonstrating that–from a first-person perspective–dementia can be seen a disorder that is related in particular to questions about selfhood, social relations and social roles. For the people involved, instead of what dementia *is*, the focus is on what Alzheimer’s disease *means* and *does* and how it affects daily life.

## Introduction

People affected by a form of dementia undergo an accumulation of progressive and chronic losses on a personal and social level [1]. Memory loss often threatens a person’s sense of security, autonomy and being a meaningful member of society [1]. Theories have been developed to explain how individuals adapt to dementia and cope with the accompanying losses [2–4]. One model that has been applied to a wide range of chronic illnesses is the ‘self-regulatory model (SRM) of illness behaviour’. SRM tries to capture the reciprocal relations between illness and behaviour [4–6] and suggests that individual differences in coping are related to different illness representations [7]. For example, Matchwick and colleagues (2013) showed how so-called ‘cause and control illness representations’ impact the way people respond to their diagnosis and try to make sense of their illness [8].

These psychological approaches deepen our knowledge about how we can facilitate application of psychosocial therapies [3] by focussing on cognitive or behavioural strategies, self-regulation, self-management and *individual* coping strategies. This focus, however, also has its limitations and may carry the risk of reducing the complexity of how people deal with the disease and the accompanying losses in at least three ways:

First, we should be aware that it is not enough to understand human nature, consciousness and meaning in terms of taxonomies, causal and probabilistic explanations. Understanding the nature and the meaning of everyday existence–and more specifically when the apparent normalcy of this everyday existence is gravely threatened by a chronic disease such as dementia–goes far beyond cause-and-effect rationality [9].

Second, it tends to psychologize and privatize, as if ‘dealing with the threat of dementia’ and ‘sense-making’ occur within an individual, and more specifically within someone’s mind [10]. Such inwardness is a simplification, as it likely overlooks that ‘dealing with’ is about people who–as bodily beings–find themselves together with others in time and space within a complex reality of social-cultural structures [10]. Experiences are always embodied and nested within a larger context [11, 12].

Third, the language of sense-making strategies, self-regulation, self-management and coping presupposes a rational, active and choosing human being. This ignores the fact that not only do people act and are capable of deciding, they also always passively *undergo* diseases, actions of other people, context and atmosphere [10]. Indeed, for a comprehensive understanding of the lifeworld of people with dementia and the way they relate to their illness, it is crucial to acknowledge this tension between activity and passivity. Dealing with the threat of dementia means “going through motions of possible uncertainty, anxiety and unexplained deterioration of his or her conditions” [10]. These more passive experiences are on a different level than actively making choices and coping with things, and therefore require a different scope and language.

Although most health professionals and health researchers are profoundly aware that good care not only requires interventions, therapies and/or treatment, but also implies that professionals foster attunement to how an individual experiences things [13], the number of robust studies that contribute to a deeper understanding of the nature and meaning of living with dementia is still limited. Experiences of living with dementia from the perspective of people with dementia have been explored in a number of valuable studies [1, 7, 14–19], but, again, the results focus mainly on psychological impact and coping strategies.

This qualitative study aims to contribute to our understanding of the day-to-day experiences by providing an idiographic description of what it *means* existentially to be in the world as a person affected by a form of dementia, trying to understand how the experiences are mediated and conditioned by the context (such as other persons, situations, events, certain places and the broader society).[11] Such an approach can assist health professionals by increasing attentive thoughtfulness–described as a minding, heeding and caring attunement–[20] which facilitates practical tactfulness, as well as a questioning mood with respect to the people concerned.

## Methods

### Context

This study is conducted in a collaboration between researchers who work in different contexts and are involved in different projects. (See S1 File for an elaboration on the context of these projects.).

### Ethical approval

For the first project, we obtained approval from the AISSR (Amsterdam Institute for Social Science Research) Ethical Advisory Board and the Faculty Ethics Committee (2014, number: 2014-AUSSR-3805). For the second, approval was obtained from the University Medical Center Groningen (2010, number: M10.094306).

### Data collection

In this study, we define dementia as a chronic decline in cognitive function that causes impairment relative to a person's previous level of social and occupational functioning [21]. It is a medical condition that can result from diverse causes, the most common of which is Alzheimer's disease. Other dementias include Lewy body dementia, frontotemporal disorders, and vascular dementia, MCI or Parkinson’s disease dementia [21]. Given our aim and phenomenologically inspired methodology, this study does not focus on differences or similarities arising from these distinct forms of dementia. Instead we attempt to grasp the overarching existential meaning of living with a form of dementia in order to facilitate a deeper empathic understanding (rather than explanation) of this experience.

We analyzed a combination of narrative accounts from people with a form of dementia, which were generated in two related projects: A large part of the data used in this present study came from the Dementia Diaries project. In the first year (from November 2016 to November 2017) sixteen people with dementia joined this project. Participants were recruited through presentations in Alzheimer Cafes by the project advisory panel, an advertisement on a digital platform and through the snowball method. They were included if they had a type of dementia, if they had sufficient verbal ability to participate in the diary project and if they were able to give informed consent. Before joining, they received information about the aim of the project. To ensure a careful informed consent procedure, both the people with dementia and a significant other gave full written permission to use their stories for our website (https://www.dementieweb.nl/verhalen/dementiedagboeken/), as well as for research purposes. On a regular basis (some more frequent than others, see Table 1), the participating diarists reflect on what it means for them to live with dementia. They use mobile handsets (customized for maximum simplicity) to record audio diary entries. They also have a contact person to assist them if necessary. For this study, we used all 322 recorded messages from the first year, which were transcribed verbatim (median 203 words per message). See Table 1 for an overview of diarists’ characteristics.

**Table 1:** Overview of participating diarists’ characteristics.

Gender	Number of diarists *(n = 16)*
Male	8
Female	8
**Year of birth**	
1930–1939	4
1940–1949	8
1950–1959	3
1960–1969	-
1970–1979	1
**Partner status**	
Living with a partner	7
Living alone	9
**Living situation**	
At home	11
Assisted living	4
Unknown	1
**Number of years since diagnosis (in 2018)**	
2 years	4
3 years	3
5 years or more	6
Unknown	3
**Type of dementia**	
Alzheimer's disease	9
Vascular dementia	4
Frontotemporal dementia	1
Parkinson's disease dementia	1
Unknown (still in the diagnosis trajectory; the diagnosis was not official yet)	1
**Number of recorded diary messages**	
up to 39	13
more that 40	3

In order to gain a deeper understanding of the complex experiences at stake, we chose to combine and complement our diary data with interview data. [22] These interviews were conducted in the context of a similar project PratenOverGezondheid Nederland [the Dutch counterpart of the Database of Individual Patient Experiences of health and illness (DIPEx)] [23–25]. Thirty-seven Dutch informants nationwide were interviewed about their experience of living with dementia. The interviews were collected using maximum variation sampling [26]. Participants were recruited through specialized case managers, support groups, GPs, day centres, the project advisory panel, advertisements, and through the snowball method. They were included if they had a type of dementia or were in a diagnostic trajectory, if a professional or informal caregiver believed that the person with dementia had sufficient verbal ability to participate in an interview and if the person with dementia was able to give informed consent. For an overview of interviewees’ characteristics, see Table 2.

**Table 2:** Overview of participating interviewees’ characteristics.

Gender	Number of interviewees *(n = 37)*
Male	17
Female	20
**Partner status**	
Partner	31
No partner	6
**Year of birth**	
1920–1929	6
1930–1939	13
1940–1949	11
1950–1959	3
1960–1969	0
1970–1979	1
Unknown	3
**Age when receiving the diagnosis**	
50–59	5
60–69	11
70–79	8
80+	9
Unknown	4
**Number of years since diagnosis**	
1 year	14
2 years	7
3 years	5
4 years	3
5 years or more	4
Unknown	4
**Type of dementia**	
Alzheimer's disease	22
Vascular dementia	3
Mixed dementia (Alzheimer and vascular dementia)	2
Frontotemporal dementia	1
Dementia with Lewy bodies	1
Mild cognitive impairment (MCI)	1
Unknown (still in the diagnosis trajectory; the diagnosis was not official yet)	7

Prior to the interview, informants received an information letter outlining aim, research procedure, privacy and contact details. Before the interview, the informant gave full written informed consent to use the interview for research purposes. The interviewer conducted face-to-face in-depth interviews in the informants’ home environments, which were audio- and videotaped with their permission. The interviews were in-depth narrative interviews, conducted with a view to uncovering experiences of dementia across a range of topics. The first part of the interview had a narrative structure in which the informants were encouraged to tell their story. Narrative interviewing was selected to enable informants to articulate their experiences, perspectives and concerns in their own way [27]. In the second part, a semi-structured interview approach was adopted to ask about topics of interest that had not been raised yet and to explore key topics in-depth. (See S2 File for the topic list). Data collection was completed when the interviews did not reveal new ideas, experiences, or insights and the data were saturated. The interviews lasted from 29 minutes to 138 minutes (median 72 minutes). Interviews were transcribed verbatim.

### Data analysis

Our iterative data analysis was inspired by Van Manen’s existential phenomenological approach.[9, 28] This approach allows for an investigation of experience as it is lived rather than conceptualized, and consists of a dynamic interplay between the following activities:

- eliciting concrete and contextualized lifeworld descriptions;
- reflecting on the essential themes which characterize the phenomenon by moving back and forth between the sense of the whole and its details;
- describing the general meaning structure of the phenomenon through the art of writing and rewriting [9, 20, 28].

The first step of the analysis was reading the complete transcripts to understand the overall meaning. The first author used Atlas.ti for MAC, version 1.5.3 to facilitate organization, coding of the data, and easy comparison of meaningful fragments and clusters. However, higher levels of interpretation occurred through our collaborative analysis and reflection by the authors [9, 28]. The significance of structuring our phenomenological description around the ‘lifeworld existentials’ of relationality (lived relationship between the self and others), embodiment (lived body), spatiality (lived space), and temporality (lived time) [9] turned out to be helpful during our analysis. In phenomenological research, these four lifeworld existentials are seen universal themes of life, arguably pervading the lifeworld of all human beings, regardless of their situatedness [9, 28–30]. They proved to be helpful guides in exploring the meaning structure of ‘living with dementia’ in terms of interpersonal relations, body, space and time. They are used in an inductive way: together they form a lens that enables to illuminate the textured, embodied, experienced world, each emphasizing particular nuances of the experience in an explorative way. (See S3 File for an elaboration on these lifeworld existentials).

A full description of a meaning structure should always be accompanied by an analysis of particularities, nuances, and unique contextual variations in the meaning of the phenomenon. It is this full structure of both generality and particularity that enhances our understanding of the issue.

### Maximizing the rigour of the study

We applied the following framework commonly used for maximizing the rigour of phenomenological studies: rigour, relevance, resonance and reflectivity [31]. To enhance rigour, we aimed for variation and information richness of cases [22, 32], resulting in a combination of in-depth interviews and longitudinal diaries. Outcomes were validated in dialogues among the research team. Besides, for a member check, the first author visited three of our most actively participating diarists to discuss the final themes, not only for validation but also for ethical reasons [31].

Regarding the value of the study in terms of its relevance, we tried to provide a rich and accurate but also contextualized description of the phenomenon. In coherence with the underlying phenomenological paradigm, the results are not perceived as universal, factual, and/or quantitatively generalizable, but rather as existential, meaning-based understandings that should provide insight into life as it is essentially lived. [28, 33]. In order to be resonant and enable the reader to engage with the people in question, the rich and vivid descriptions should help the reader to actually feel what it might be like to live with dementia. It is an attempt to find the ‘I in the Thou’ [29]. In addition to being true to the phenomenological approach, this also aligns with our deep conviction that people with dementia are people like ourselves, sharing full humanity; thus, it is an attempt to go beyond stigmatization and medicalization. In line with the phenomenological approach, the main purpose of our member check (with the three most active diarists) was not about agreement, but rather about whether the outcomes of the study resonated with participants’ experiences [30, 33]. In terms of reflectivity we have aimed for an attitude of openness, susceptibility and sensitivity to the participants and to the phenomenon during the whole research process.

## Results

In this section, we describe the experiences of living with a form of dementia from a first-person perspective. The relationship with one’s own body, with others and with the surrounding world is severely disrupted because of the dementia. People with dementia have to face all kinds of losses and difficulties, but we found that the attitude of others is what has most impact on daily life. Below, we first provide a thematic overview (Box 1).

**Box 1. Overview of main themes**

**‘The eyes of others’ are what really matters**

Disrupted body

- Scrutinizing your disrupted body
- Trying to control your bodily loss-of-control

Disrupted relationships

- Feeling scrutinized by the suspicious gaze of others
- Drifting away from significant others
- Having difficulties sharing the struggle
- Longing to be taken seriously
- Engaging in a world of peers

Disrupted sense of space and time

- Sensing disorientation in an alien place
- Feeling closed in within a shrinking space
- Trying to control a dreaded future
- Trying to control a dreaded future by means of euthanasia

### Disrupted body

#### Scrutinizing your disrupted body

From the moment participants started noticing that something was seriously wrong, they became much more aware of their body, so clearly and movingly expressed by one of the diarists:

This morning, I stood in front of the mirror and–while looking at my face, closely observing it–I thought: nothing has really changed yet. You know, I don’t have that glazed, staring look you sometimes see in people with dementia. In contrast though, I pondered, within my head, the dementia has definitely changed everything! It’s like a kind of grey veil has fallen over your brain and I really want to clean it up. I’ll do my best, but clearing up doesn’t seem to work. Talking about it, however, does, so I'm trying to be happy about that. (V40)

Participants started scrutinizing their familiar body for its unusual behaviour, clumsy gestures, inappropriate language and uncontrolled mind. It increasingly felt like an alien body on which they can no longer rely, controlled by unfamiliar, foreign forces. The body they had always relied on now showed signs of betraying them. Against their will, the dementia took charge, and their body is becoming a constant source of loss and disruption. Paradoxically, this strange body is also undeniably their own. There is no other option than to somehow relate to it.

Participants often experienced the diagnostic tests in the hospital as exams which they are doomed to fail. Most realized that the tests are quite easy and yet too difficult for them, which confronted them even more with their deteriorating body, strengthening feelings of failure and loss:

At the start of the test, I thought: Well, this really is a piece of cake. The questions and sums were so silly, so obvious! But at a certain point, *(starts crying)* I noticed that it was not clear to me at all …(DEM18)

After receiving the diagnosis, many participants found themselves navigating between shock and relief. Suddenly, their suspicions were confirmed. There is no way back, their bodily state will never improve, it will only deteriorate. What awaits them is an unclear but gravely threatening future. About his worrying, one man said:

After receiving the diagnosis, I was constantly thinking of it. In fact, I often lay awake all night, which was really an attack on my body, but even more on my mind. You know, if I cannot sleep, lots of things go through my mind. It’s one large teeming snake pit then, this head of mine, a teeming snake pit. At that moment nothing is good, everything is negative …(V158)

While the ‘verdict’ is often experienced as terrible, for many it is also a relief. Many participants had become preoccupied with a bodily sense that something was seriously wrong. Now that their problem was defined medically, their strange behaviour could be ‘explained’. When confronted with disturbing symptoms, at least they could now tell themselves: 'This is what it is. This is what I have to live with.' In a way, the diagnosis allowed them to fit their disrupted bodies into their life. Besides, the diagnosis gave them access to professional health care and support systems. The outcomes of the diagnostic process, however, did not always correspond with people’s daily experiences. Sometimes, the body seemed to 'say' something different than the scans. In such cases, the diagnosis could be experienced as quite confusing:

Last week I visited the geriatrician (…). It turned out that my whole brain is affected by the…, well…, in fact, I don’t exactly know how to put it. Actually, they were completely white … So, it seems that I have quite a widespread form… I, however, tend to think that–when I evaluate my daily functioning–it’s actually not too bad yet. I’m still able to manage a lot all by myself. So in the end, I have no idea how to interpret the diagnosis… But I cannot change it, that’s for sure.(V195)

While some expressed their scepticism about the insufficient medical knowledge on dementia, others experienced the medical language mainly as alienating, a language they could not understand, as if it is not about them or their bodies.

It is a process, of course, with all kinds of concepts (…) going right over my head. Many Latin concepts, you know, when it comes to the pills and the treatment. And there is little support to build up a new story. I’d prefer it if they said: it states 'blah blah, but this is what it means’. But that is not what happens …(DEM02)

#### Trying to control your bodily loss-of-control

For most, dealing with ongoing loss-experiences and deterioration was a very hard process. Many participants were never again at ease with their body. It generated uncertainty not only about *what* they could *do* but also *who* they *are*. Their whole bodily existence was profoundly put into question by the suspected signs of dementia. Some participants no longer dared to trust their perception and became quite uncertain about their own bodily performance. Confronted with their physical limitations, they tried to control the symptoms by feigning normalcy, and hiding the strangeness.

I used to engage in conversations with other people but now I sometimes notice that I don’t give the right answers, you know, stray from the theme. That makes me terribly insecure. I can’t trust myself. (…) Right now, I’m scrutinizing my choice of words, but still I say crazy things. It makes me think: ‘You stupid fool!’ (…) Most people are very decent, they’ll never joke about it (…) but I have this annoying feeling that I'm not equal anymore. I’m different…(DEM37)

Most participants wanted to stay involved in life as much as possible, but they felt thwarted in their ambition by the limitations of body and mind:

I went to Dave [son] to solve a software problem in a program I developed myself for him in the past. Before I left I had a pain in my stomach. I really dreaded going there. Could I postpone it…? That would perhaps be the easiest way, but it seemed unsatisfactory. Luckily, Dave was not as chaotic as he can be, and he did not fly into a rage like the last time. That made it more pleasant. But unfortunately, after much trying, I didn’t succeed in solving the problems for him. I was very disappointed. Again, it was confirmation that I don’t function well any longer.(V118)

All kinds of bodily symptoms and complaints were related to dementia, such as stumbling, difficulty riding a bicycle, sleep problems, but also intestinal complaints, diabetes and heart failure. Participants told us that they do not want to ‘attribute everything to dementia’. Simultaneously, though, in their search for explanation and causality, they tried to regain control of a highly uneasy process and get a grip on the bodily losses.

### Disrupted relations

#### Feeling scrutinized by the suspicious gaze of others

Participants scrutinized themselves, but they also feel scrutinized by the gaze of others. The diagnostic process was about locating dementia in their bodies, as well as within their social relations. With the diagnosis, all kinds of conceptualizations and stigmatizations of dementia emerged and reshaped other people’s ideas and interpretations about the new situation. For many, this created deep uncertainty about the interpersonal contact with others and their social position: Can I still be myself? How do others view me? Do others immediately recognize that something is ‘wrong’? Am I still considered worthy and capable of doing things? In many cases, participants immediately sensed a change in the relationship with others:

If you are diagnosed with dementia, to others it sounds like, all of a sudden, you are assumed to be incapable of anything. But actually, that’s ridiculous (..) You know, I have lots of experience, and that’s not all gone at once.(DEM15)

Well, you're going one step down in society. You’re no longer considered a full member of society. And in discussions you’re also a ‘quantité negligable’. Someone who is now avoided by everyone.(DEM37)

Most participants expressed a longing for a shared journey, but often felt quite alone; being thrown back upon their own resources. Sometimes they even exacerbated the loneliness by masking or hiding manifestations of dementia from others, in an attempt to avoid the risk of judgment and stigmatization. In many cases, participants explicitly mentioned that ‘the sense of being looked at’ was what troubled them most.

#### Longing to be taken seriously

Several participants were upset by the interventions of others. Well-intentioned but often unsolicited, people tended to take over certain tasks: deciding that they were no longer able to work, drive a car, or participate in a hobby club. In some cases, there was even reluctance on the part of spouses when their loved one wanted to join the dementia diary project, because the spouses feared failure. A side effect of this attitude was that participants frequently felt patronized. ‘Their restrictions are imposed on me’ and ‘more and more, my freedom is taken away’. It is experienced as a dehumanizing experience. Instead of having a sense of ‘us together’, they now felt like it was ‘them against me’. One lady told us about her family purchasing an induction plate for her ‘behind her back’:

I really don’t think it was necessary yet; it was still safe for me to cook. But they didn’t trust me anymore. The bad thing is: it’s decided for me, but I have to pay for the new plate! (…) I really have trouble with the fact that things are forced on me.(V1)

Another participant said that, after the diagnosis, he really wanted to continue working on a therapeutic basis, but unfortunately, that did not work out. His colleagues could not handle it. He was told that ‘he drove them mad’. It strengthened his feelings that ‘everything is slipping away’. Another man was frustrated that, due to dementia, he was not even considered eligible for volunteer work, while he felt that he still could be of use to the organization. For many, it felt as if the diagnosis immediately degraded them to ‘second-class citizens’; all serious responsibilities were taken away by others.

Most participants were aware of the increasing limitations and losses they had to face. Yet they wanted to be directly involved in how to deal with those limitations. For example, one man dealt with the painful loss of his driving licence by transferring his insurance to his wife. For him, doing this transfer himself was a way to ‘stay in charge’. Other participants spoke about choice making regarding their next holiday. They themselves decided that a holiday abroad might be too distracting, and therefore opted for a domestic vacation. Others chose not to organize huge birthday parties or wedding anniversaries anymore, preferring a celebration in a small, familiar group. Although these decisions in themselves were often painful, taking preventative measures to reduce the effects of dementia, redrawing their own boundaries and setting out new preconditions gave participants a sense of self-worth and mastery, even in the context of loss.

While a sense of not-being-taken-seriously-anymore prevailed in the accounts, participants also told stories about feeling respected. One man, for example, expressed his deep appreciation about still being ‘tolerated’ as a ‘respected member’ of a board, being allowed to chair the board meeting. Another man was still involved in the checkers club, and even joined the competitions. While they kept in mind that at some point they would have to stop, they felt they were not ready to do so.

Within the context of the shrinking social environment, competences and possibilities, several participants succeeded in finding other/new ways to express themselves. One diarist posted two beautiful sound messages on which she played the piano (instead of talking about her daily life) as if saying: ‘This is me as well, this is how I express myself best.’ Others joined artistic initiatives such as ‘DemenTalent’ (http://dementalent.nl) or a dance performance ‘Brain Shadows’. In terms of the valuation of activities that were specifically organized for people with dementia, several participants stressed that they valued the challenge to learn new things (instead of ‘being kept busy’). Some started writing short articles for the support group magazine, others developed new skills, such as painting or sculpting or writing poems. Some diarists explicitly noted that joining our research project gave them a sense of meaning. One participant suggested that we should rename our dementia diaries and call them ‘Brain Power Diaries’, which reflects the importance he attached to his expertise.

#### Having difficulties sharing the struggle

Many participants had difficulty sharing their sadness or frustration about their struggle with the disease with their network. Friends and acquaintances seemed to keep more and more distance from the person with dementia.

As far as relationships are concerned, I just notified everyone what the diagnosis was, but nobody responded, except someone who exclaimed: I’m so sorry for you! But, eh, no one has asked me what it really means for us, for our relationship. So yes, I really feel a bit disappointed, because [through my exposure] I felt very vulnerable, but I did not get a single reaction.(V140)

People had trouble sympathizing. They tend to look away, try to cover it up with well-intentioned but relativizing comments or platitudinous remarks like: “Luckily it’s not too bad yet.” One participant, who reflected on her feelings of not being appreciated, put it this way:

Well, sometimes it's not fun at all to have vascular dementia. Oh man, there are moments I climb the walls. I just had a call from a friend, and you know, because of this illness, I sometimes have a short fuse. People just don’t understand. The same goes for this friend, she's covering it up, cloaking it in silence in a way that makes me want to vomit. Then I think, damn, just let me finish my story without trying to avoid the issue (…) She just leaves me with my frustrations.(V48)

Although friend stayed away in quite a few cases, there were also many people who felt generously supported by their network. In some cases, the network was even enlarged and strengthened. However, even good support from the network did not always take away feelings of helplessness and hopelessness:

We are happy with the support we have from all sides now. Really…, really… But I still feel hopeless. Presumably, you can hear it in the tone of my voice, both helpless and hopeless.(V242)

The support was often practical. Sharing the emotional or existential struggle seemed much harder, both for the person with dementia and for the significant other.

#### Drifting away from significant others

The support of close family members or friends had become indispensable, as they put things in perspective and provide much needed guidance in an uncertain world. For most participants, this support was both a source of gratitude and deep concern. Many respondents were haunted by the uneasy feeling of being a burden to their network. Some felt so deeply dependent on their partner that they did not dare to go anywhere without them, being very worried about the possibility of losing their partner. “Then I’ll be completely lost, nowhere, you know (…) those are things that are going through your mind all the time, and that’s a highly unpleasant feeling, I can tell you."

Slowly but surely participants felt they drifted away from their significant others. They talked about thinking on different levels, living in different worlds, assessing things differently. They were unable to keep up with things adequately. Many came to the sad conclusion that they do not feel like equals anymore. For some, the reality of a compromised relationship due to the dementia created a deep feeling of uncertainty and inequality. In their experience, the disease caused tension with respect to intimacy.

The daily communication with my partner is becoming a problem. To put it like this: if you draw two lines, her line continues on the same level, but mine deflects in a downward curve. Clearly, our communication no longer runs in two parallel lines. As a result, I tend to withdraw. I don’t really want to isolate myself, but actually, that’s what I do. I also feel that, at times, I don’t have much to contribute anymore. More than before, I hesitate to ask or discuss things with her. That’s a rotten side-effect of dementia.(V177)

Several people explicitly talked about a negative polarization of roles in relationships. From the diagnosis onwards, previous roles such as spouse, father, employee or friend were in many cases unwittingly replaced by being a ‘care-receiver’ first and foremost. Instead of being together and taking care of each other, the persons diagnosed with dementia experienced themselves as ‘solely the one being cared for’. Gradually, the relationship became more imbalanced and one-sided, losing a sense of mutual complementarity, responsibility and reciprocity. While many participants were very aware of the fact that they were unable to cope with life without help, they also had a strong wish to continue to contribute in other ways, but often they felt without value in many aspects of life.

I've been at the centre of many kinds of things that I've been involved in for a long time, but now, it's exactly the other way around. Right now, I’m just set aside so to speak.(DEM14)

Clearly, tension in relationships was present in most accounts. There were, however, also various stories about improved relationships, especially with close family members. Participants talked about deepening the relationship from a cognitive to a more emotional level, enjoying meaningful moments more intensely, appreciating little things more fully, and feeling safe and able to surrender yourself to your loved ones. One man talked about his relationship with his partner’s grandchildren towards whom he had always felt somewhat inhibited:

The relationship with my granddaughter suddenly became so much better. I opened up a bit more to her and immediately I got very nice feedback: ‘hey grandpa Danny’. And eh, for me as a childless figure, that’s a very pleasant experience. Apparently, I didn’t lose the ability to love my grandchildren, well my partner’s grandchildren. And that's just really nice.(V164)

#### Engaging in a world of peers

For many respondents, contact with people in the same position was experienced as a relief. Not because they talked about living with the disease and exchanged advice, but mostly because they did not need to talk about it. There was what they called ‘unspoken understanding’ or ‘a kindred spirit’. Participants spoke about other people with dementia in terms of ‘dementia colleagues’, ‘dementia comrades’, ‘dementia supporters’ or ‘fellow members of the dementia club’. They shared the sense that they were ‘kind of in the same boat’. One man–who joined a support group called ‘The Boosters’–said:

It’s just a group of people who have also been told that they have Alzheimer's. One is in a more advanced stage than the other, but I feel comfortable. We value and fully respect each other. We laugh, talk, drink coffee, and we do nice things.(V2)

Most participants felt at ease amongst others of their kind, who were perceived as peers. ‘It’s just the relaxed atmosphere, you know, I can just be myself.’ Participants especially valued that–among peers–they did not have to ‘hide’ the symptoms of the disease. In ‘normal life’, some felt that people trivialized the impact of the problems they faced. For them, it was a relief to be acknowledged and taken seriously by their peers:

I'm so glad I got in touch with them. For me, those are the days I feel good. When we are together, we can just be ourselves and I don’t have to defend myself each time against people who downplay it and say: 'Oh, don’t you pay it any mind, I forget so much myself'.(V134)

Whereas a sense of reciprocity was often decreasing in many aspects of life, the support group for most participants strengthened the feeling of being involved and being able to provide help or advantages to each other. For example, by encouraging each other to stay active:

One of our fellows, the youngest one, is now recovering from a recent stroke. Since this occurrence, we always go and pick him up at his clinic on Thursdays and take him with us in a wheelchair. And then we go for a good walk, and we visit a museum. We have done that two or three times already, and he looks forward to it.(V22)

While participants were at ease and felt ‘normal’ when they were with so-called ‘like-minded people’, it also made them sad. One man talked about his decision to go on a multi-day trip for people with dementia. On the one hand, he really enjoyed the time together and perceived it as a relief that he ‘didn’t have to fight all the time (…) Things just ran much smoother’. But it also led him to a ‘deeply sorrowful conclusion’ about the relationship with his beloved wife. In his words: ‘I've come to the realization that the differences between the two of us have become unbridgeable.’

### Disrupted sense of space and time

#### Losing grip on the basic familiarities of life

Most participants recounted the experience of becoming strangers in their own world; they often feel disorientated or lost. Gradually, the world becomes an increasingly alien place. The feeling of basic familiarity diminishes. Meaningful connections between the self and the outside space are interrupted, creating feelings of not-being-at-home and insecurity. Due to the dementia, life gradually loses its coherence and continuity. Their pattern of daily life is disarranged. Often the natural rhythm of time, of day and night is disrupted. In many cases, their untimely, forgetful or repetitive behaviour made participants feel quite uncomfortable.

Sometimes I’m in one of my moods, and I am worried sick about the future, not being able to organize things, occasionally taking the wrong bus or forgetting to get off, failing to remember street names, searching for words … But most times, I manage to handle myself … I try to stay active and keep doing as much as possible with the help of my iPhone and iPad.(V5)

Feeling unable to change the dementia, they instead made adjustments in their lives and houses to try to counter this sense of estrangement in terms of time and space. They added objects and tools (such as clocks, calendars and schedules on the wall full of memos, or iPads) to their lives in an effort to orientate themselves, maintain grip and “remember” the daily routine.

In the beginning, I had lots of trouble determining what day it was, and eh, which date. But they designed special clocks for us, so I bought one. In big letters, it shows me that it’s Friday, December 2, and I can read it when I sit at my desk, working with the computer. So, I'm on time, and up-to-date again.(V8)

My iPhone is becoming more and more a concatenation of memories and appointments. It beeps all day long. Without it, little would come of anything.(V5)

Other participants talked about the importance of going beyond the shame and asking for help, thereby increasing freedom and space.

I like cycling (…) but I have to pay attention, because this is a forest area. So one time I got lost and couldn’t remember anything. But, then, I went to a house, rang the doorbell, and introduced myself–who I am, and that I have Alzheimer’s–and I asked whether they could help me. [laughs]. Usually, though, I can find my way because we have a big church here (…) with a very tall tower. So I just look for it and then I know exactly where I am.(DEM20)

Participants realized that their pace is not the same as before. They fall behind their loved ones. Although some strongly resisted it, others seemed able to accept it:

When I see others, being busy and doing lots of things, easily remembering everything they need to do, then I think: I used to do that as well, but I can’t anymore. I have to write everything down. Everything goes much slower now. (…) Do I suffer because of that? No, not really. I notice it and I'm irritated for a moment, but then I tell myself: it's just part of life, that's the price you pay for a long life, for being old. I can’t change it.(V138)

#### Feeling closed in within a shrinking space

Not only is the world becoming more unfamiliar and less like home, it is also getting smaller. Participants constantly had to adjust to more limited horizons. They talked about deliberately creating this limitedness by withdrawing from a wider social community into a close circle of intimate family and friends, thereby seeking the safety of familiar and comfortable surroundings. Other times, the outside world was also restricted by others who take over their place, and determine the boundaries for them.

This year we started to have dinner at one of us, once a month. But when it was my turn, I found my neighbour–who is my charlady and my friend as well–in my kitchen. She had already prepared everything, everything was ready. You know, she had already been in contact with the others, about how to organize it all. I was incredibly surprised, I was …, it was well-intentioned, but you know …, the annoying thing is that, that whole group has now judged, decided–without me–that I’m no longer able oversee it all. Yes, well-intentioned, but you know…(V9)

Yet mostly it was just the inevitable consequences of the advancing disease that reduced the possibilities for pursuing individual projects, hobbies or other leisure activities. In an attempt to avoid (further) hassle, participants often felt forced by the circumstances to put an end to those activities.

Recently, I unsubscribed myself from the Chamber of Commerce. That is another step on my way to a complete dead end…(V137)

After deep reflection, we decided that our way of going on holiday is no longer possible. In addition to the usual problems with me, the journey by train has become too burdensome for us. (…) Regrettably, another piece of our freedom is lost and that’s on me…(V209)

Not only the physical, outside space is changing, many participants also experienced mental distance. They reflected on an aching sense of ‘existential outsideness’, a feeling of no longer belonging in the same space. Even while loved ones were physically near, mentally the distance was getting bigger and bigger, leaving them in a limited and separated space:

I'm afraid tonight I don’t have a very positive message. Actually, I’m really suffering due to the influence of dementia. You know, I have a lot of problems with the growing distance between me and my relatives. With my wife, and also with my brother-in-law and my sister who live nearby… They just go on with their lives, and I cannot keep up with them any longer. I know it’s just one of the consequences of dementia, this distance between you and the ones you love. (…) but it makes me very sad.(V298)

#### Trying to control a dreaded future

Receiving a diagnosis had a strong effect on the meaning people attached to time. On the one hand, the diagnosis came with a relative clarity: a very uncertain and disruptive period of not-knowing has come to an end. On the other hand, the diagnosis was also experienced as a new rupture, and the start of an even more uncertain and ambiguous period of life. There is no clarity about the progression of the disease, how long it will take, and where it might bring them. Mostly people were very worried about possible scenarios. Not so much the increasingly tangible proximity of the end of life, but rather the fear of mental deterioration, loss of decorum and eventually fading into oblivion.

The fear about the future considerably changed the experience of the ‘now’. Participants’ accounts are full of references to the awareness of the limited time span of still being able to enjoy their relationships, family life and holidays. They became very apprehensive about the temporality of daily life. Living in the ‘now’ constantly alternated with ‘looking ahead’ to the future that is approaching. While some were able to face this uncertain future–or even talked about ‘acceptance’–, other participants found it very difficult to deal with this realization. They tried to live on and let it happen, but their attempts were steadily overshadowed by a tense fear of the future.

Nothing could be taken for granted anymore. Some diarists were engaged in an inner dialogue about whether their assessments are still realistic, making adjustments, rethinking their future plans and rearranging priorities. Participants often contemplated the idea of decreasing possibilities and the lack of a meaningful and welcoming future. For some, this resulted in melancholy, a remembering yourself the way you were in the past, when you could still trust yourself. It also resulted in active future planning:

I have asked my geriatrician for advice, as I want to be eligible for ‘The Guest House’, which is small-scale housing for demented people that will be established here in the near future. I am going to an information evening about this next Tuesday.(V77)

#### Trying to control a dreaded future by means of euthanasia

Several participants anticipated the feared future by making a request for euthanasia. This may be related to the specific Dutch context of this research project in which euthanasia in case of dementia is an option (See Box 2 for background information). A few respondents said they wanted euthanasia in the short term, some wanted euthanasia in the future if they had to move into a nursing home, while others remained relatively vague about the timing of their wishes. Basically, the wish regarding euthanasia was driven by uncertainty and anxiety about an assumed future. Those who had an advanced directive for euthanasia often perceived it as a form of security. It seemed that having a choice (euthanasia) gave them a sense of retaining or regaining some control over dreaded future time:

When it comes to a worthy and dignified end of life, my keyword is self-determination. I want to end my life as a rational, sane person. At the moment, my thinking abilities are already a little compromised, I have to admit, but I really want to stay in control till the end of my life.(V187)

In most cases, however, the thought of euthanasia also gave rise to many dilemmas, such as gradual shifts in the wish to die over time, disagreements between those involved, and difficulty determining the appropriate moment. With regard to their euthanasia request, several respondents told us about a tension between the lived now and the feared future. Often against the odds, they still enjoyed daily life on the whole, but they were so afraid of the future that it constantly affected the lived now. One respondent went to see her GP every three months to confirm her wish for euthanasia, just to be sure, but on the other hand she expressed her surprise and gratitude for all beautiful days.

**Box 2. Background information about euthanasia in the Netherlands**

**Euthanasia in cases of dementia**

In 2002 euthanasia was legalized in the Netherlands under strict requirements laid down in the Termination of Life on Request and Assisted Suicide (Review Procedures) Act. Legal requirements of due care for performing euthanasia in the Dutch context are that:

1. the physician is satisfied that the request by the patient is voluntary and well considered
2. the patient’s suffering is lasting and unbearable
3. the physician has informed the patient about their situation and prospects
4. the physician agrees with the patient that there is no reasonable alternative
5. the physician has consulted at least one other independent physician who has seen the patient
6. the physician has exercised due care in terminating the patient’s life or assisting in the patient’s suicide

A growing group within the Dutch public is making euthanasia requests. Requests regarding the ‘foreseeable future’ increased from 13,400 per year (in 2011) to 17,900 (in 2016). Requests ‘in due time’ almost doubled, from 33,900 (in 2011) to 67,700 (in 2016).[34] Part of those requests ‘in due time’ are based on the fear of dementia, a disappearing identity and eventually the fear of an ‘untimely and undignified death’.

One of the criteria for lawful euthanasia is that the physician should be fully convinced that the request is the patient’s actual wish. Therefore, being competent and able to engage in a meaningful communication is seen as a crucial condition. In case people are no longer capable of expressing their wishes, there is also a law-based possibility to request euthanasia based on an advanced directive, which is a written statement of their preferences regarding decisions about euthanasia should they become incompetent. Consequently, people diagnosed with dementia (on their own or a relative’s initiative) increasingly want to talk about an advanced directive for euthanasia ‘in due time’ in an early phase of dementia.

It should, however, also be noted that many Dutch physicians are reluctant to perform euthanasia in cases of dementia. In the Dutch legal context, euthanasia is never a person’s right: people are free to ask for euthanasia, but the actual decision is always based on the individual and collegial considerations of the physicians involved.

Thus there seemed to be a substantial difference between the ‘right’ to choose and the actual ‘content’ of the choice. On the one hand, the fact in itself that participants had a right to die at a self-chosen moment provided them some relief and assurance. On the other hand, deciding on the *how*, *when* and/or *why* also turned out to be a quite demanding existential struggle. The legal right-to-die provided no directions for their emotional state and moral attitude.

## Discussion

This study seeks to deepen our understanding of what it means existentially to be in the world as a person with dementia. The results demonstrate that living with dementia can be understood as an ongoing, severely disruptive experience: the people concerned enter a very uncertain, unpredictable and ambiguous period of life. They have to face all kinds of losses that considerably change their relationships with others and the surrounding world. As the disease develops, social relations constantly shift and should be realigned again and again in order to accommodate the new reality. Also, the relationship with themselves and their body changes significantly. The familiar and trusted body–often unconsciously and silently performing in the background [35]–is now very much in the foreground as an alien body, controlled by unfamiliar forces, they can no longer rely on. They often find themselves confronted by the very ‘otherness’ of their bodies [36]. The body is ‘taking over, displaying a life of its own’, and yet it is their own. This is a highly unsettling and ambiguous experience, threatening a sense of intactness [35–37].

### The eyes of others

The most obvious finding to emerge from our analysis is that ‘the eyes of others’ are of enormous importance. The impact of the reaction of others to the diagnosis; the desire to still be taken seriously and be recognized; the attempt to avoid disapproval; the increasing tendency to withdraw: all these themes were implicitly or explicitly related to this bottom-line theme. In some cases, ‘the eyes of others’ created a safe and accepting environment in which the person with dementia might find ways to relate to the disease, but more often people seemed to feel scrutinized by an inquisitive and disapproving view. This objectifying gaze had a severe impact on daily life, as in several cases it provoked a sense of being patronized. They felt they were no longer taken seriously in an equal, reciprocal relationship, and in some cases even felt they were no longer approached as a ‘complete human being’. Our results suggest that this sense of marginalization and alienation is evoked, not so much by harsh, explicit words, but mainly as a result of tacit, insensitive practices and/or actions.

### Interplay between experiences and social imaginaries

Our outcomes also reveal a connection between people’s experiences of dementia on the one hand and dominant social imaginaries on the other hand (which we define as: often implicit shared notions and images of certain socio-cultural groups, involving moral claims about the basic values of society, that guide the ways in which members of a socio-cultural community imagine their existence [38–40]). Although experiences of dementia may be very personal, they do not arise in a vacuum. Indeed, personal experiences of dementia are nested within a socio-cultural environment: the meaning of these experiences is framed and conditioned in interaction with this context [11]. In our research, this interplay was reflected in the following ways:

First, as phenomenological researchers have already described, after receiving the diagnosis, people’s experiences become ‘scientized experiences’ [41]. In our contemporary culture, abstract scientific knowledge is highly valued. With the medical diagnosis, objectifying knowledge is intermingled with subjective, daily experiences. The diagnosis calls something into being, but the way this ‘new reality’ is put into words often creates distance from, and sometimes even contrasts with immediate bodily experiences [41, 42]. Our research accords with these earlier observations, and indicates that diagnostics may lead to healing clarity as well as disrupting estrangement [41, 42], or both at the same time.

Second, when affected by dementia, the experiences of the people concerned are shaped by prevailing ‘social imaginaries’. Gilliard and Higgs, among others, have thoroughly described how our collective image of aging is defined by negativity and otherness [43], and that the rise of dementia has created an even more fearful, abject image of old age [44]. According to Taylor, this negative picture derives from a society in which we regard cognitive capacities (such as memory, language and cognition) “as the necessary foundation of individual identity, personhood, and the capacity to relate to others” [45]. And Post states that ‘the ethos of selfhood is one of hyper-cognitivity that privileges rationality as the seat of the self’ [46]. These dominant perceptions result in negative labelling that ‘contributes to social abandonment and isolation of the person living under the description of dementia’. Our study clearly illustrates that much of the suffering related to dementia indeed stems from living under the shadow of these negative social imaginaries.

Third, the experienced and internalized disapproval should not be understood as a one-way process that is evoked only by the scrutinizing view of others. Giddens (1991) already demonstrated convincingly that the self is not a passive entity, determined by external influences. On the contrary. He argued that individuals also contribute to and directly promote certain social influences [47]. Personal and social spheres should thus be seen as fluidly interwoven spheres [38, 47]. Consequently, social imaginaries on dementia can be perceived as ‘lived spaces’ in which people share, shape and challenge the meaning of their existence [39, 40, 48]. Consistent with this theory, we found that participants constantly scrutinized themselves with a criticizing and judging view, influenced by negative, defining societal imaginaries. Simultaneously, by participating in our research (the Dementia Diary Project and PratenOverGezondheid, the Dutch DIPEx) they make comparable, but also contrasting experiences of dementia public, which may lead to a nuancing or even a shift in existing public views.

### Practical implications

The central strength of this study lies in the fact that it is one of the few in-depth studies that give a voice to people diagnosed with dementia. To date, these voices often remain unheard and insufficiently acknowledged. This might lead to misalignment, both in research and practice [49]. For health professionals, it is essential to take into account human, embodied experience in all its complexities. By providing empathic insight into precisely these complex experiences, our study may foster a deeper understanding of the illness, its impact on people’s lives, and its existential meaning [50]. A phenomenological approach to dementia is promising in that it goes beyond the prevailing reductionist and objectifying view [51, 52]. Indeed, it provides a non-judgmental tool [50] that allows a receptive view which is characterized by openness towards the world and the needs of the other” [51] [52].

As shown by the study, disruption of social relationships and roles is a great burden for dementia patients. While ageing scholars have been pleading to take into account the social context of dementia for many years now [53–56], the main focus in research so far is still on the biomedical and psychological approaches of dementia [57]. Our study supports the demand for a socio-relational approach by demonstrating that–from a first-person perspective–dementia can be seen as a disorder that is related in particular to questions about selfhood, social relations and social roles. Our study also underlines the importance of appropriate social interventions for people with dementia, and confirm the idea of Johnston et al. that such interventions should seek to enhance personhood, facilitate meaningful engagement and offer the potential for the person to leave a legacy [58]. Rather than what dementia *is* (e.g. a medical condition, clinical diagnosis, disrupted bodily functions, something that must be cured), for the people involved the focus is on what the disease *means* and *does*, for example, that it deeply affects their ability to continue their lives, relations and activities.

The outcomes of our study thus provide further support for the thesis that research and medical practice are perhaps still too preoccupied with causes and symptoms, overlooking the meaning for the persons involved. Based on our research [59–61], we conclude that substantive change in the current focus is necessary. This is not, however, a plea for a new dichotomy. Conversely, dementia should be seen neither as a cognitive dysfunction nor a socio-relational construct only. Instead, to do justice to the complex reality of living with dementia, our research and practice should include both sides.

### Limitation of the study

There are some considerations that should be taken into account when interpreting our results: First, following from our aim to grasp the overall existential meaning of living with a form of dementia, we deliberately chose to include people with different forms of dementia, in different stages of the disease. The member check we conducted demonstrated that the described bottom line of this study strongly resonated with participants’ experiences.

The fact remains, however, that different people, different forms of dementia and different contexts will result in unique experiences and emphases. Researchers and practitioners should therefore always strive for a balance between a shared, general understanding of the experience of living with dementia and a unique, personal understanding of this specific case [22, 62].

Second, our findings are somewhat limited by the fact that joining our study (and our projects the Dementia Diary Project and the Dutch DIPEx) requires certain cognitive linguistic skills. While some diarists were assisted by their loved ones, our approach unavoidably excluded those who are not able to express themselves through talking or writing. This is an important issue for future research. To develop a full picture of what it means to live with dementia, additional qualitative studies will be needed that shed light on the experiences of those who are unable to join common interviews or keep a diary.

Third, this study was conducted in The Netherlands; a high-income Western-European country with an advanced and sophisticated system of dementia diagnosis and care. In addition, it is a country that has the option of euthanasia. This specific context certainly influences the outcomes, and the results may not be directly generalized to the lived experience of people living with dementia in other socio-cultural and economic environments. This is, however, typical for qualitative phenomenological outcomes; they should never be seen as final and absolute outcomes. While phenomenological research strives to describe the essential meaning of the phenomenon under research, it is also clear that meaning should always be viewed as contextual and infinite.[22, 28, 33] In the context of this study, generalization should thus not be understood as universal, factual, and/or quantitative generalization, but as an ‘existential generalization’ [28] which means ‘a meaning-based, essential understanding of the phenomenon’ that should help to ‘make contact with life as it is lived’[28].

## Supporting information

S1 File Context information about the projects. (DOCX)

S2 File Topic list interviews. (DOC)

S3 File Elaboration on the lifeworld existentials. (DOCX)
