# Supplementary material for: ‘The eyes of others’ are what really matters: The experience of living with dementia from an insider perspective
Source: PLoS One. 2019 Apr 3;14(4):e0214724. doi: 10.1371/journal.pone.0214724 (PMC6447241; doi:10.1371/journal.pone.0214724)
Supplement: S1 File — (DOCX) [file pone.0214724.s001.docx]

**Appendix 1 – Context information of this research project**

**In this Appendix, we provide some additional context information about the two projects involved in current study.**

***The Dutch Narratives of Dementia Collection and the Dementia Diaries***

The last author (AMT) is an anthropologist and professor in Long Term Care and Dementia at the University of Amsterdam. For many years, she conducted research into daily life of people with dementia (1-3). **In January 2015, AMT launched the *Dementieverhalenbank,* which is** a Dutch digital Narratives of Dementia Collection, in order to **preserve and share the narratives of people with dementia and their caregivers. Since November 2016, the Dementieverhalenbank is extended by the Dementia Diaries project that collects people’s various experiences about living with dementia as a series of audio diaries and presents them on a website:** <http://dementieverhalenbank.nl/dementiedagboeken/>**.**

**With the** Narratives of Dementia Collection**, we have several aims, namely: 1)** to **teach** the value of deep listening**; 2) to share supportive experiences for those living with the condition; and 3) by** increasing the visibility of the situation at home, to **nuance and** differentiate the dominant (sometimes disastrous) societal images of dementia **– and weave into the fabric of our society an understanding of the daily life of people living with dementia**. To this end a selection of interviews was edited into readable narratives that were anonymously published on the website ([www.dementieverhalenbank.nl](http://www.dementieverhalenbank.nl)) and in a book (4).

**Most importantly, the fourth and main objective of the** Narratives of Dementia Collection **is to** create a valuable archive for research purposes. By analyzing the stories, we aim to grasp the (often hidden) needs in the everyday life of people living with dementia. This provides insight into what it means to live with dementia for those diagnosed with the disease and their significant others. These insights are used to improve the care and support for people with dementia and their **significant others, and to develop new interventions (2, 5).**

Synchronous with the launch of the digital narrative collection, the *Proeftuin Sociale Benadering Dementie* was established, which is a unique experimental learning and research laboratory for the structural development, implementation and evaluation of new ideas, interventions, methods and institutional arrangements in dementia care. The main aim of this laboratory is to enable a better life for people with dementia and their close ones (2). Today, the laboratory has developed into a broad and unique collaboration between the research institute *Tao of Care*, a major healthcare institute (De KwadrantGroep), case managers, GPs, gerontologists and health insurers (6). Based on research and in co-creation with the care professionals and policy makers, AMT developed ‘The Social Approach to Dementia’ (6, 7), which is a practice-based approach that seeks to continuously improve the daily life of people with dementia and their networks.

***The Dutch DIPEx website PratenOverGezondheid – Talking about Health***

The original idea for a ‘Database’ of Individual Patients’ Experiences (DIPEx) came from Oxford GP Dr Ann McPherson and Dr Andrew Herxheimer, clinical pharmacologist. Dr Ann McPherson had been diagnosed with breast cancer and although she knew about the medical side, she couldn’t find anyone to talk to about what it was really like to have this disease. This and Dr Herxheimer’s experience of knee replacement surgery, prompted them to come up with a website that would provide reliable information about ordinary people’s experiences of health and illness: healthalk.org.

Healthtalk provides free, reliable information about health issues, by sharing people’s experiences. This website aims 1) to support patients and their loved ones, who may feel alone or ill-prepared for challenges ahead; 2) to support healthcare professionals in providing patient-focused care; 3) to promote better communication between patients and health professionals.

To ensure that the information on the website is balanced and accurate, a rigorous qualitative research method developed by the Health Experiences Research Group of the Oxford University is used (8-10). The aim of this method is to interview enough people (between 30 and 50) from different backgrounds that: 1) the information on the website represents the full range of experiences that might be connected with a health conditions; 2) visitors to the website could find experiences that they relate to; and 3) the information provided help people to make informed decisions about their health, backed up by solid evidence. The main findings from a study are published in 20-30 detailed thematic summaries on the website, illustrated with hundreds of video clips. Each project is supported by an advisory panel made up of people affected by the health issue, health professionals, academics and staff from relevant patient charities.

This qualitative research methodology has been taught to partners in universities in other countries. All these universities are collaborating in the DIPEx International network, which aims to improve the understanding worldwide of personal health experiences for the benefit of our communities, researchers and the health sector, including healthcare professionals and policy makers.

PratenOverGezondheid is the Dutch counterpart website set up in the Netherlands by the University Medical Center Groningen. The second author (MA) has been involved in this project since the start in 2011 and represents PratenOverGezondheid in the board of DIPEx International. At the moment, the PratenOverGezondheid website covers sections on dementia – from the perspective of the person with dementia themselves as well as the caregiver - on diabetes type 2 and on chronic kidney diseases. In addition, sections on rare diseases, primary care for children, and pregnancy and childbirth are in progress. Through our research we provide evidence-based data on people’s experiences of health and illness which offers information and support, and assists informed decision-making.

**References:**

1. The AM. In de wachtkamer van de dood: Leven en sterven met dementie in een verkleurende samenleving. Amsterdam: Thoeris; 2005.

2. The AM. Dagelijks leven met dementie: een blik achter de voordeur [Daily life with dementia: looking behind the front door]. Amsterdam: Thoeris; 2017.

3. The AM. Herken de mens met dementie: zoeken naar een balans tussen cure and care in een palliatieve zorgpraktijk. Zwolle: Christelijke Hogeschool Windesheim; 2011.

4. The AM, Roolvink A, Smit M, editors. Van verhalen naar inzichten: Een selectie uit de Dementie Verhalenbank. Amsterdam: Dementie Verhalenbank; 2015.

5. van der Wedden H, Komen R, Van der Reijden I, Henning Z, The AM. Van verhalen naar inzichten: Eerste rapportage van de Dementieverhalenbank in opdracht van het Ministerie van VWS. Amsterdam: Tao of Care; 2017.

6. The AM, Jonkers R. Zaaien en oogsten: Proeftuin Sociale Benadering Dementie. Amsterdam: Tao of Care; 2017.

7. The AM, Kooij S, Planting A, editors. Sociale Benadering Dementie: Het beste medicijn. Amsterdam: Dementie Verhalenbank; 2015.

8. Herxheimer A, Ziebland S. The DIPEx project: collecting personal experiences of illness and health care. Narrative research in health and illness. 2004:115-31.

9. Herxheimer A, McPherson A, Miller R, Shepperd S, Yaphe J, Ziebland S. Database of patients' experiences (DIPEx): a multi-media approach to sharing experiences and information. The Lancet. 2000;355(9214):1540-3.

10. Ziebland S, McPherson A. Making sense of qualitative data analysis: an introduction with illustrations from DIPEx (personal experiences of health and illness). Medical education. 2006;40(5):405-14.
