# Supplementary material for: ‘The eyes of others’ are what really matters: The experience of living with dementia from an insider perspective
Source: PLoS One. 2019 Apr 3;14(4):e0214724. doi: 10.1371/journal.pone.0214724 (PMC6447241; doi:10.1371/journal.pone.0214724)
Supplement: S2 File — (DOC) [file pone.0214724.s002.doc]

**Topic list interviews people with dementia**

1. ***Preparations***
   - Answering questions
   - Signing Informed Consent
   - Preparing and testing audio and video recorder
2. ***First part interview – narrative interview***
   - Could you please tell me your story from the first moment you noted that there was something wrong?
3. ***Second part interview – semi-structured interview***

*Before the diagnosis*

1. First signs of dementia:

- Could you tell me how you noted that something was wrong?
- What kind of changes in your memory have you noted?
- When did you encounter the first problems?
- How did you explain these changes/problems?
- What did you do when you had the feeling that there was something wrong?

1. Referral:

- By whom were you referred to the medical doctor in the hospital?
- How did you perceive the moment you were referred to the hospital?

1. Diagnostic test

- How did you perceive undergoing the diagnostic tests in the hospital?
- What do you think of the information and support you got from professionals when undergoing the diagnostic tests?

*Receiving the diagnosis*

1. Diagnosis dementia
   1. *The moment of getting the diagnosis*

- Can you describe the moment when you heard that you have a type of the dementia?
- How did you react?
- What kind of (professional) support did you get at that moment?
  1. *Familiarity with dementia*
- What did the diagnosis dementia mean to you?
- What type of information did you search for? What did you need at that moment?
- What do you think of the provision of information? Was it sufficient? Did you miss anything?
  1. *Reaction of family and friends*
- How did your family and friend react when they heard that you have dementia?
- How did you perceive this reaction?

*After the diagnosis*

1. Living with dementia
2. *Coping with dementia*

- How do you live with the dementia?
- Can you explain to me what it means to live with dementia?

1. *Impact of dementia on the daily life*

- Can you describe how your daily life looks like now?
- Your daily life
- Your family life
- Your social life
- What has changed in your daily life since the diagnosis?
- What do these changes mean to you?
- How do you cope with these changes?
- What is difficult in your life with dementia?

1. *Making life bearable*

- What helps to make your life with dementia bearable?
- What is the role of your philosophy of life/religion/spirituality in your life with dementia?

1. Person with dementia

- Can you tell me how you as a person was before the dementia and how this differs from the person now?
- How do you perceive these changes?
- Can you tell me how your social environment treats you?

1. Care

- From whom do you get help in your daily life?
  - Informal caregivers
  - Health care professionals
  - Family
  - Friends
- How do you perceive this help?
- How do you perceive the help from health care professionals?
  - GP
  - Medical doctor
  - Case manager
  - Nurses
  - Professionals at day care organisation

*The future*

1. View of the future

- How do you see the future?
- To what extent do you prepare for the future already?

1. ***Closing interview***

- What would be your advice to other people who face dementia?
- Is there anything else you would like to share?
