# Supplementary material for: ‘The eyes of others’ are what really matters: The experience of living with dementia from an insider perspective
Source: PLoS One. 2019 Apr 3;14(4):e0214724. doi: 10.1371/journal.pone.0214724 (PMC6447241; doi:10.1371/journal.pone.0214724)
Supplement: S3 File — (DOCX) [file pone.0214724.s003.docx]

Appendix 3_ An explanation of the four fundamental lifeworld existentials.

- The lifeworld existential **‘relationality’** was used to reflect on the question how the situation affects the relation between self and others. How is the self-experienced in relation? How is the interpersonal contact experienced differently from the contact before the diagnosis?
- The lifeworld existential **‘embodiment’** was used to reflect on the question how the situation affects the way the body is experienced. Is it experienced as an object of a subject? How is the situation felt in the body, in its gestures? How is the body of the self and other perceived differently?
- The lifeworld existential **‘spatiality’** was used to reflect on the question how the experience of space is affected by the situation. How is the personal topography affected by the situation? What is the meaning of distance and closeness and how do changes in spatiality affect lived experience?
- The lifeworld existential **‘temporality’** was used to question how the meaning of time is affected by the situation. How do people’s experience (objective and subjective) time, the past and the future, continuities and discontinuities?

**References:**

1. van Manen M. Phenomenology of practice: Meaning-giving methods in phenomenological research and writing. Walnut Creek, California: Left Coast Press; 2014, pp. 101-105

2. van Manen M. Researching lived experience: Human science for an action sensitive pedagogy. Ontario: State University of New York Press; 1990, pp. 302-306
